# The Epitope and Neutralization Mechanism of AVFluIgG01, a Broad-Reactive Human Monoclonal Antibody against H5N1 Influenza Virus

**DOI:** 10.1371/journal.pone.0038126

**Published:** 2012-05-25

**Authors:** Zhiliang Cao, Jiazi Meng, Xingxing Li, Ruiping Wu, Yanxin Huang, Yuxian He

**Affiliations:** 1 MOH Key Laboratory of Systems Biology of Pathogens, Institute of Pathogen Biology, Chinese Academy of Medical Sciences and Peking Union Medical College, Beijing, China; 2 Wenzhou Medical College, Wenzhou, China; 3 National Engineering Laboratory for Druggable Gene and Protein Screening, Northern Normal University, Changchun, China; Shanghai Medical College, Fudan University, China

## Abstract

The continued spread of highly pathogenic avian influenza (HPAI) H5N1 virus underscores the importance of effective antiviral approaches. AVFluIgG01 is a potent and broad-reactive H5N1-neutralizing human monoclonal antibody (mAb) showing great potential for use either for therapeutic purposes or as a basis of vaccine development, but its antigenic epitope and neutralization mechanism have not been finely characterized. In this study, we first demonstrated that AVFluIgG01 targets a novel conformation-dependent epitope in the globular head region of H5N1 hemagglutinin (HA). By selecting mimotopes from a random peptide library in combination with computational algorithms and site-directed mutagenesis, the epitope was mapped to three conserved discontinuous sites (I-III) that are located closely at the three-dimensional structure of HA. Further, we found that this HA1-specific human mAb can efficiently block both virus-receptor binding and post-attachment steps, while its Fab fragment exerts the post-attachment inhibition only. Consistently, AVFluIgG01 could inhibit HA-mediated cell-cell membrane fusion at a dose-dependent manner and block the acquisition of pH-induced protease sensitivity. These results suggest a neutralization mechanism of AVFluIgG01 by simultaneously blocking viral attachment to the receptors on host cells and interfering with HA conformational rearrangements associated with membrane fusion. The presented data provide critical information for developing novel antiviral therapeutics and vaccines against HPAI H5N1 virus.

## Introduction

Highly pathogenic avian influenza (HPAI) H5N1 viruses continue to spread among poultry and have frequently broken the species barrier and transmitted to humans. As of February 2012, there were 583 confirmed human H5N1 infections from 15 countries, with a fatality rate of >59% (344), alarming that the consequences of a H5N1 pandemic could be catastrophic. Therefore, many efforts have focused on the development of effective therapeutics and vaccines in preparedness. Of them the neutralizing antibody-based strategies have been particularly explored.

The viral hemaglutinin (HA) surface glycoprotein of influenza A viruses is not only responsible for binding to cell receptor but also a primary target of neutralizing antibodies. It is initially synthesized as a precursor polypeptide (HA0) and subsequently cleaved by cellular proteases into disulfide-linked HA1 and HA2 subunits. The N-terminal HA1 subunit forms a globular head region that contains the receptor-binding site (RBS), whereas the membrane anchoring HA2 subunit forms a helix-rich stem that contains a relatively conserved fusion peptide. The HA protein of influenza A viruses evolves with great genetic diversity and can be classified into 16 distinct subtypes. However, few of the 16 subtypes have been finely characterized with respect to their antigenic structures. In the early 1980s, the location and structure of HA epitopes was first characterized in the three-dimensional (3D) model of the H3 subtype [1]. Four antigenic sites were demonstrated (A, B, C, and D), and a fifth (E) was later described. The H3 structure was used to map the antigenic sites of H1 [2], H2 [3], and H5 [4] subtypes. The H5 HA was antigenically mapped in greater detail after its crystal structure was reported [5]–[7]. Prominently, the antibody binding epitopes of the H5 HA are located exclusively in areas corresponding to antigenic sites A and B of H3 HA and the antigenic site Sa of H1 HA. Recently, Kaverin *et al* [8] demonstrated that the HA antigenic structure of recent HPAI H5N1 isolates differs substantially from that of low-pathogenicity H5 strains described earlier and is rapidly evolving.

It is also noteworthy that previous epitope mapping of HA protein were largely based on the virus escape mutants selected by mouse monoclonal antibodies (mAbs), which might not precisely predict the antigenic epitopes recognized by human immune system [9]. The apparent success of passive immunotherapy of a H5N1-infected patient with convalescent plasma emphasizes the importance of human antibodies in fighting against influenza [10]–[11], and also sparks the development of neutralizing human mAbs as antiviral agents by various approaches. Importantly, human mAbs can also be used to map the antigenic epitopes of HA that stimulate humoral immune responses during the natural infection of influenza viruses and thus serve as an ideal basis for vaccine development. Recently, Sun *et al* generated two H5N1-neutralizing human mAbs, AVFluIgG01 and AVFluIgG03, by screening a phage display library derived from a recovered patient infected with highly pathogenic H5N1 viruses [12]. Previous studies showed that AVFluIgG01 had a broad-spectrum anti-H5N1 activity and its passive immunization could efficiently protect mice from a lethal H5N1 virus infection [12]. In this study, we have focused to characterize its antigenic epitope and neutralization mechanism. Our data have demonstrated that AVFluIgG01 targets a novel and conserved conformation-dependent epitope located in the globular head region of HA and exerts its neutralizing activity by simultaneously blocking viral attachment to the cell receptors and interfering with HA conformational rearrangements associated with membrane fusion.

## Results

### AVFluIgG01 Recognizes a Conformational Epitope within HA1

**Figure 1:**
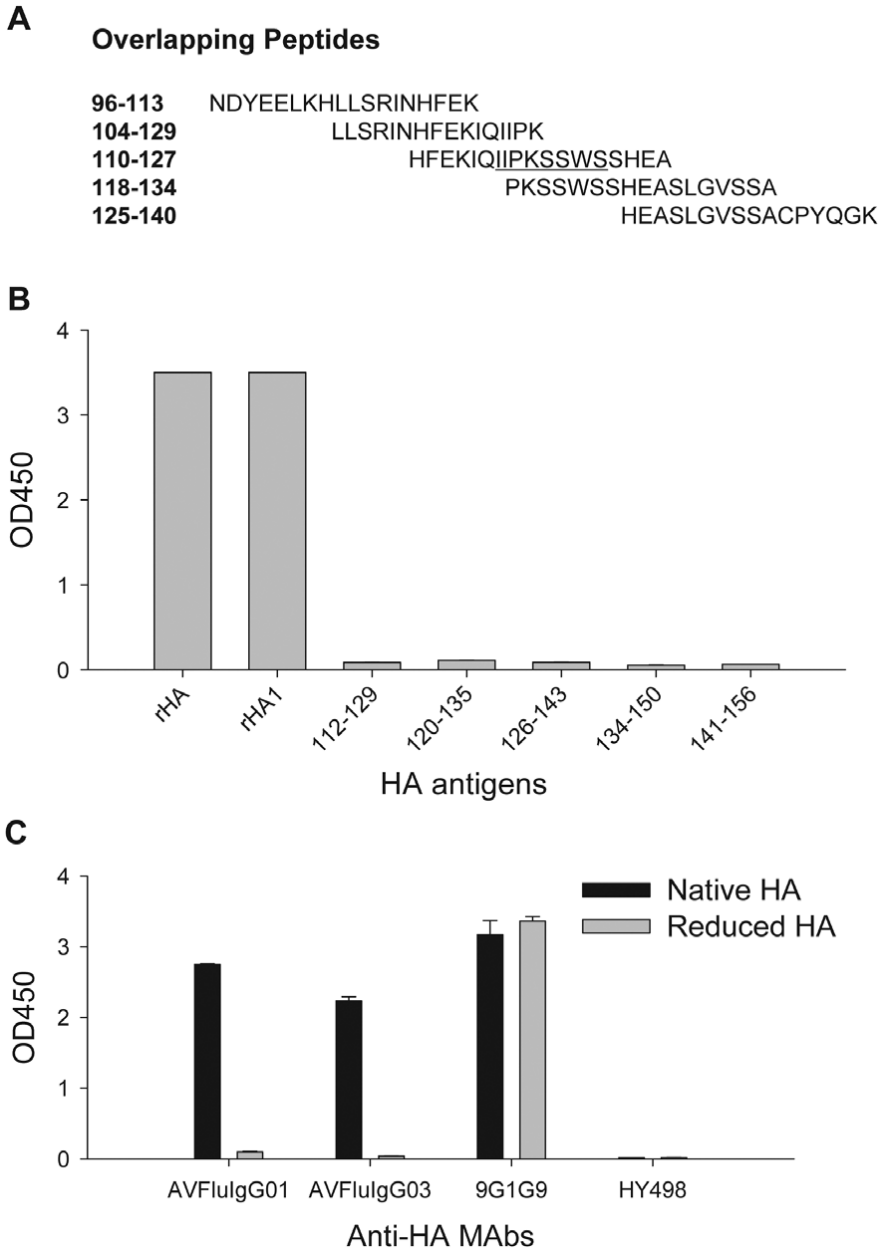
Reactivity of AVFluIgG01 with different HA antigens. **A**. Representative sequences of synthesized HA peptides (Viet04). **B**. Reactivity of AVFluIgG01 with the recombinant HA or HA1 proteins (Viet04) and overlapping peptides in ELISA. **C**. Reactivity of AVFluIgG01 with the native and reduced HA proteins (HK06) in ELISA. Synthesized peptides at 5 µg/ml or recombinant HA proteins at 1 µg/ml were used to coat 96-well microtiter plates, and AVFluIgG01 or a control mAb was tested at a final concentration of 10 µg/ml.

Previous studies concluded that human mAb AVFluIgG01 targets a linear epitope within a sequence of the ^116^IIPKSSWSS^124^ in the global head region of HA [12]. To better locate its epitope residues, we synthesized a set of overlapping peptides that cover full length HA and used in peptide-based ELISA (Fig. 1). Disappointedly, AVFluIgG01 did not react with the peptide 110–127 that contains the ^116^IIPKSSWSS^124^ sequence and any of other overlapping and non-overlapping peptides, while it reacted strongly with recombinant HA and HA1 proteins (Fig. 1A–1B). This result implied that the epitope for AVFluIgG01 could not be simply located within the ^116^IIPKSSWSS^124^ motif. Therefore, we tested the reactivity of AVFluIgG01 with a DTT-reduced HA in comparison with the native HA. Fig. 1C shows that disulfide bond reduction of HA protein could completely abolish the binding of AVFluIgG01. Serving as a control antibody recognizing a conformation-dependent epitope, AVFluIgG03 reacted with the native but not reduced HA similarly. Severing as a control antibody recognizing a linear epitope within HA2, 9G1G9 reacted with both native and reduced HA proteins. These results indicated that AVFluIgG01 was directed against a disulfide bond-dependent conformational epitope expressed on the HA1 subunit.

### Computational Prediction of AVFluIgG01 Epitope

**Figure 2:**
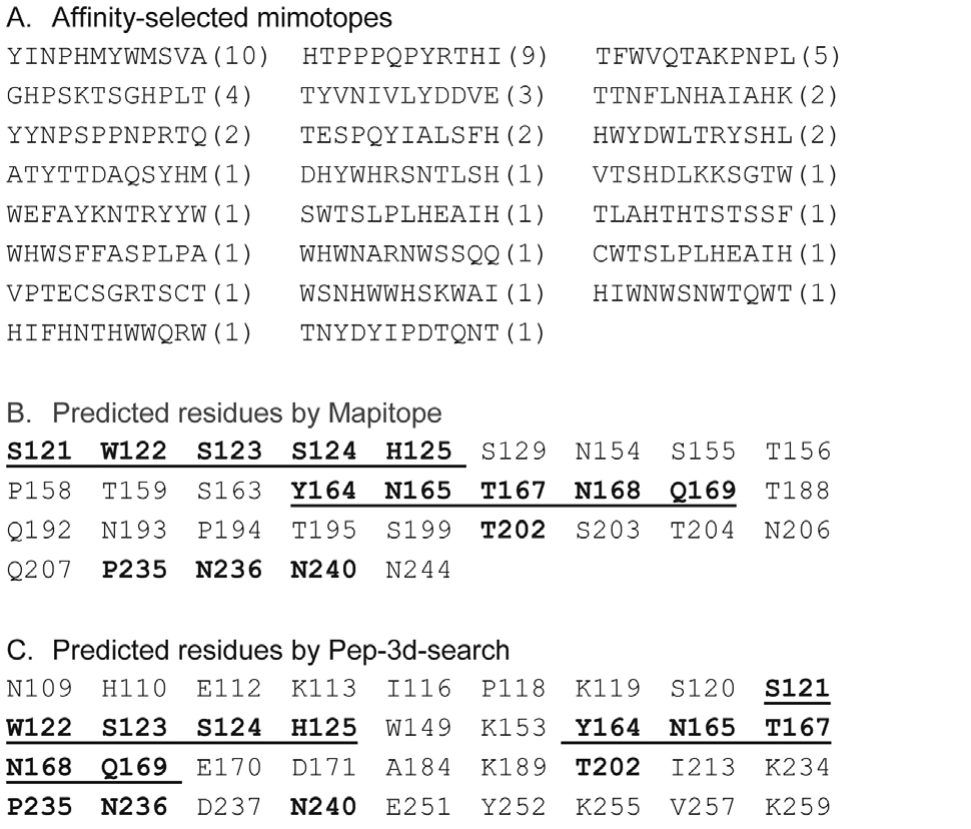
Prediction of AVFluIgG01 epitope by minotopes. **A**. Affinity-selected minotopes from a random peptide phage display library. The selection frequency of a specific minotope is shown in parentheses. **B**. Predicted amino acid residues by Mapitope. **C**. Predicted amino acid residues by Pep-3D-Search. The common residues that are predicted by both algorithms are underlined and shown in bold.

We sought to decipher the binding sites of AVFluIgG01 by selecting mimotopes from a random peptide phage display library, as previously used to map conformation-dependent mAbs [13]–[14]. After 3 rounds of panning, 53 phage clones were isolated and the amino acid sequences of their peptides were determined (Fig. 2A). The specificity and relative affinity of the binding of these peptides to AVFluIgG01 were tested in ELISA as described. From total of selected 23 different mimotopes, we could not find apparent consensus sequences or common sequence motifs that can deduce epitopic HA residues. Therefore, we used two computational methods, Mapitope and Pep-3D-Search, to predict the AVFluIgG01 epitope. Both searching algorithms are preferentially for identifying discontinuous B-cell epitopes based on the 3D structure of a protein antigen [15]–[16]. As shown in Fig. 2B–2C, the Mapitope predicted a total 32 residues while the Pep-3D-Search predicted 36 residues. All predicted residues are exclusively located in the global head region of HA1 subunit. Notably, two patches of residues (S121, W122, S123, S124, H125 and Y164, N165, T167, N168, Q169) were predicted by both programs, suggesting an involvement of these two sites for AVFluIgG01 binding.

### Identification of the Epitope Residues by Mutagenesis

**Figure 3:**
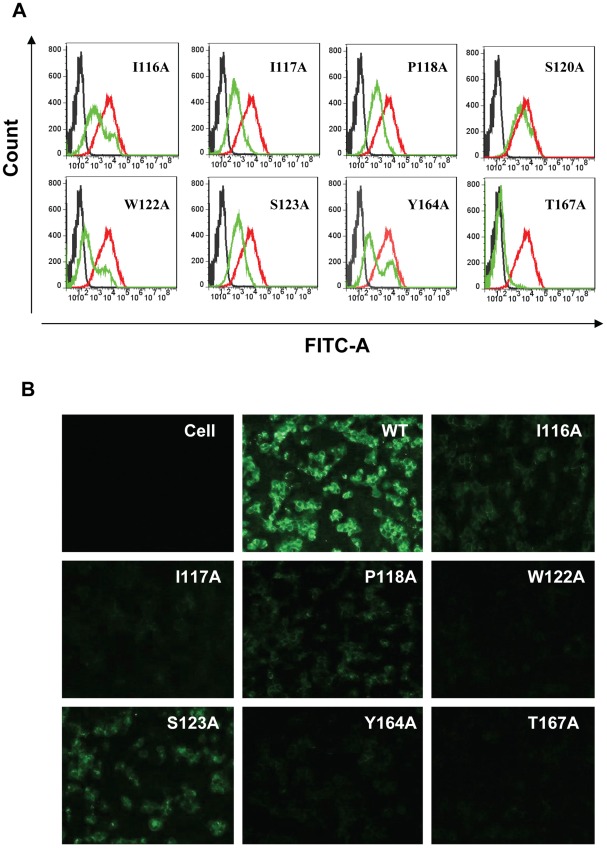
Mutagenesis analyses of the predicted epitope residues. **A**. The reactivity of AVFluIgG01 with wild-type (WT) HA or HA containing site-directed mutations analyzed by flow cytometry. **B**. The reactivity of AVFluIgG01 with the WT (red curve) or HA carrying single mutations (green curve) analyzed by immuofluorescence assay (IFA). In both assays, the WT HA or mutants were transiently expressed on 293T cells by transfection and then immunostanined by AVFluIgG01 or a control mAb at a final concentration of 10 µg/ml.

To identify the epitope residues critical for AVFluIgG01 binding, we generated a panel of 30 HA mutants carrying single amino acid substitutions (Table 1). The wild-type (WT) and mutated HA proteins were transiently expressed on the surface of 293T cells by transfection. The reactivity of AVFluIgG01 with each HA mutant was examined by IFA and FACS assays. The results are shown in Table 1 and Fig. 3. As a control for expression, no mutations affected the binding ability of a human mAb directed against the HA2 subunit. Prominently, substitutions of W122 and T167 with alanine completely abolished the binding of AVFluIgG01 to the HA expressed on the cell surface, while substitutions of the residues I116, I117, P118, S123, and Y164 resulted in a dramatic loss of the reactivity. This result verified these residues as critical sites for AVFluIgG01 binding. In addition, mutations at N165 and N168 also substantially reduced the reactivity of AVFluIgG01 with the expressed HA in the FACS assay. The remaining substitutions had no significant effects on the binding of both AVFluIgG01 and control antibodies.

**Table 1:** Reactivity of AVFluIgG01 with HA mutants.*

	AVFluIgG01		Control mAb	
HA	IFA	FACS	IFA	FACS
Cells	–	–	–	–
WT	+++	+++	+++	+++
N109A	+++	+++	+++	+++
H110A	+++	+++	+++	+++
E112A	+++	+++	+++	+++
K113A	+++	+++	+++	+++
**I116A**	+	+	+++	+++
**I117A**	+	+	+++	+++
**P118A**	+	+	+++	+++
S120A	+++	++	+++	+++
S121A	+++	+++	+++	+++
**W122A**	–	–	+++	+++
**S123A**	+	+	+++	+++
H125A	+++	++	+++	+++
S155A	+++	+++	+++	+++
T156A	+++	+++	+++	+++
S163A	+++	+++	+++	+++
**Y164A**	+	+	+++	+++
N165A	+++	+	+++	+++
**T167A**	–	–	+++	+++
N168A	+++	+	+++	+++
Q169A	+++	++	+++	+++
Q192A	+++	+++	+++	+++
N193A	+++	+++	+++	+++
P194A	+++	+++	+++	+++
S199A	+++	+++	+++	+++
T202A	+++	++	+++	+++
S203A	+++	+++	+++	+++
T204A	+++	+++	+++	+++
Q207A	+++	+++	+++	+++
N236A	+++	++	+++	+++

* The reactivity of AVFluIgG01 with HA and its mutants was tested at 10 µg/ml by IFA and FACS respectively. “−” indicates negative reaction; “+, ++ and +++” indicate weak, medium, and strong positive, respectively. The bold mutants indicate the residues critical for AVFluIgG01 binding.

**Figure 4:**
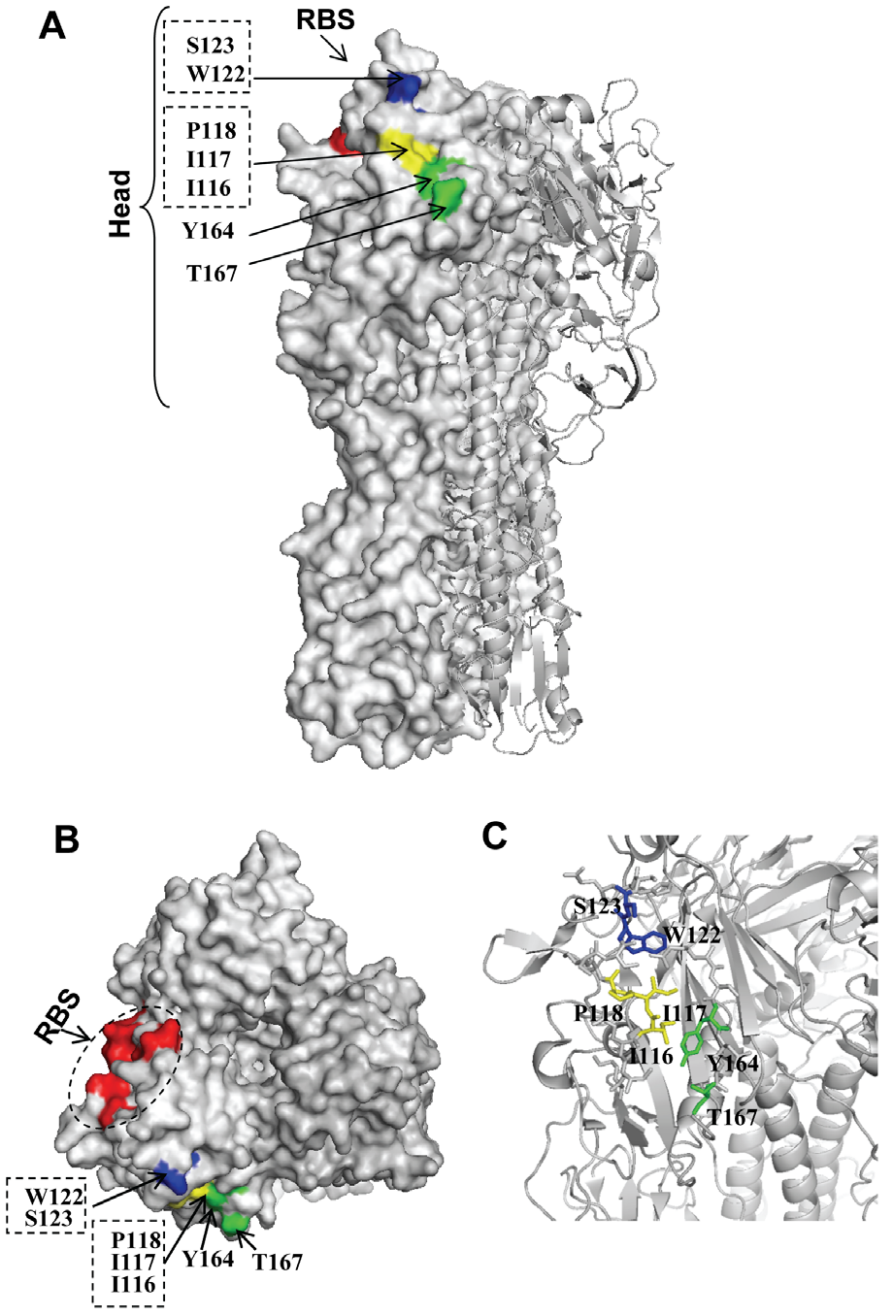
Surface representation of the AVFluIgG01 epitope on the globular head of HA trimer model (PDB ID: 2IBX). Amino acid positions are designated in H5 numbering. **A**. Side view of the trimeric HA structure. The epitopic site I residues (I116, I117, P118) is colored in yellow, and site II (W122, S123) and site III (Y164, T167) residues are colored in blue and green, respectively. **B**. Top view of the globular head. The region encompassing the receptor binding site (RBS) is colored in red. **C**. Cartoon and sticks illustration of the three-dimensional structure of the AVFluIgG01 epitope. The diagram was generated with PyMOL.

Therefore, three discontinuous sites, designated site I (I116, I117, P118), site II (W122, S123), and site III (Y164, T167) are likely involved in the AVFluIgG01 epitope. In the 3D structure, the epitope residues are adjacent to each other at the tip of the membrane-distal globular domain of HA monomer, in close proximity to the receptor-binding domain (Fig. 4). Importantly, these amino acids are highly conserved among diverse H5N1 clades and subclades [12]–[13], [17], underlying the broad-spectrum H5N1-neutralizing activity by AVFluIgG01.

### AVFluIgG01 Inhibits Both Virus Attachment and Post-attachment Steps

**Figure 5:**
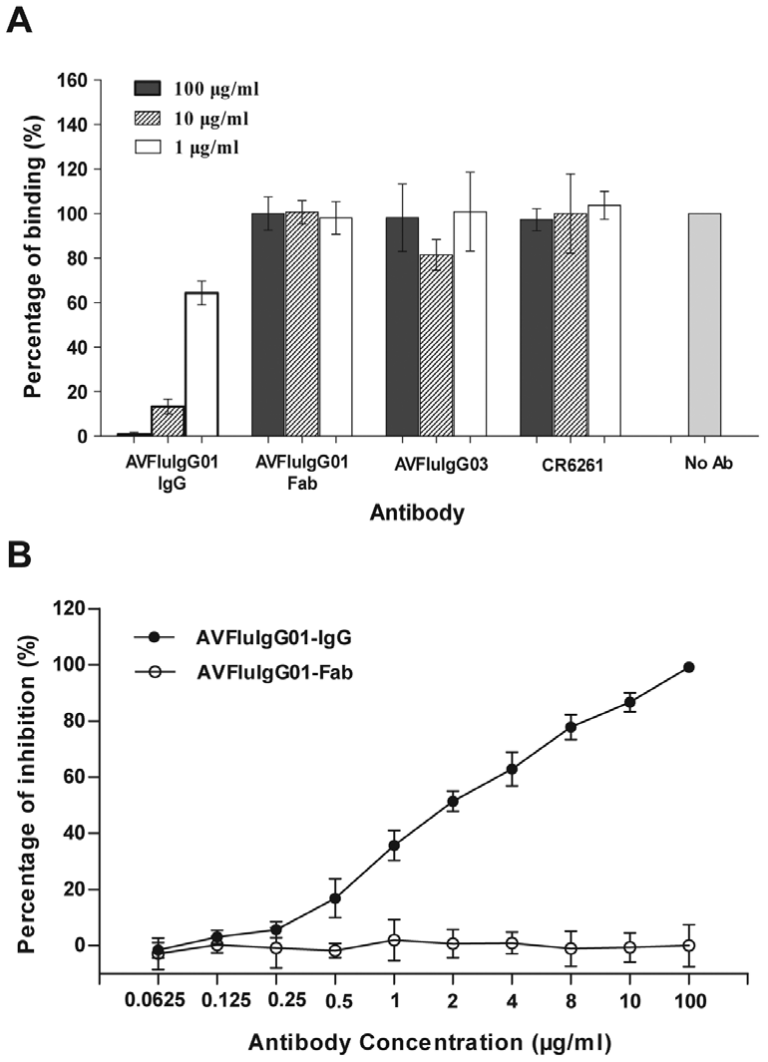
Inhibition of AVFluIgG01 on HA-mediated virus-receptor binding. The Viet04 HA pseudoviruses were preincubated with the tested antibodies at 4°C overnight and subsequently added to MDCK cells for 2 hours at 4°C. The unbound viruses were washed off and the amount of bound viruses was quantified. Binding was expressed as a percentage of the reading obtained in the absence of antibody (No Ab), which was set at 100%. **A**. AVFluIgG01 but not its Fab fragment or control mAbs (AVFluIgG03 and CR6261) inhibits the pseudovirus binding to MDCK cells at indicated concentrations. **B**. AVFluIgG01 but not its Fab fragment inhibits the pseudovirus binding at a dose-dependent manner.

Cell entry of influenza A viruses is mediated by its HA protein through two steps [18]. First, the HA protein binds to the cell receptor resulting in the endocytosis of virus into the host. Second, the viral membrane fuses with the endosomal membrane to release the viral RNA into the host cell. To explore the mechanism of neutralization by AVFluIgG01, we first performed experiments to know whether this mAb can inhibit the receptor-binding step. As shown in Fig. 5A, the virus binding inhibition assay indicated that AVFluIgG01 could efficiently inhibit HA-mediated virus binding to the MDCK cells. Apparently, this inhibition was at a dose-dependent manner (high, medium, and low). Fig. 5B shows an inhibition curve with IC_50_ at 1.85 µg/ml. However, the Fab fragment of AVFluIgG01 had no such inhibitory activity. As expected, two control mAbs, AVFluIgG03 and CR6261 could not inhibit the virus-receptor binding. It is known that AVFluIgG03 has no reactivity with the tested HA (A/H5N1/Vietnam/04) and CR6261 targets a conserved epitope in the HA2 subunit [12], [19].

**Figure 6:**
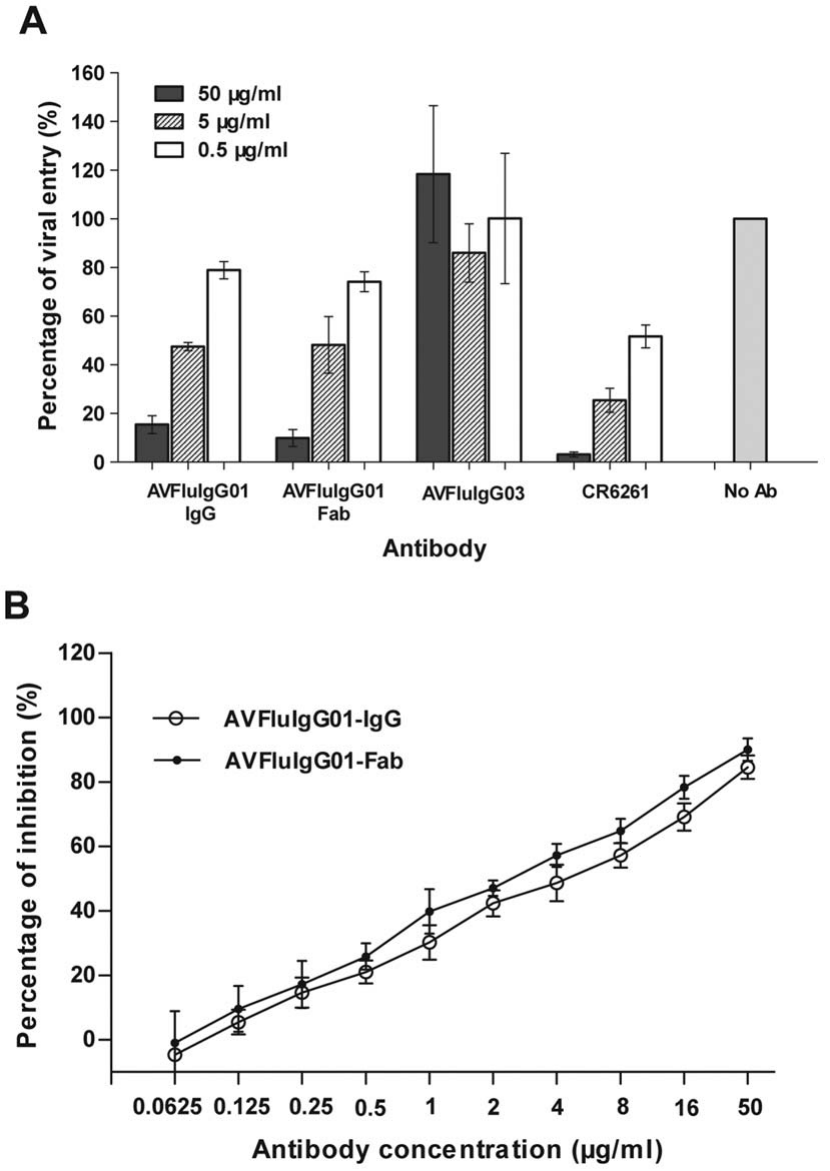
Postattachment neutralization of AVFluIgG01 and its Fab fragment. The Viet04 HA pseudoviruses were allowed to attach to monolayer MDCK cells at 4°C for 6 hours. The unbound viruses were washed off and the tested antibodies were added to the monolayer for 2 hours at 4°C. Then, the cells were incubated at 37°C for 72 hours. The luciferase activity in the cell lysates was determined. Viral entry was expressed as a percentage of the reading obtained in the absence of antibody (No Ab), which was set at 100%. **A**. AVFluIgG01 and its Fab as well as CR6261 inhibit the viral entry at indicated concentrations. **B**. Both of AVFluIgG01 and its Fab inhibit the pseudovirus entry at a dose-dependent manner.

Further, we studied whether AVFluIgG01 can inhibit the virus after its attachment on the target cells. In the postattachment neutralization assay, the HA pseudoviruses were allowed to absorb to MDCK cells at 4°C and the unbound viruses were washed off. Different concentrations of antibodies were then added to determine if they can prevent the attached virus from entering the target cells by membrane fusion. As shown in Fig. 6, both IgG and Fab molecules of AVFluIgG01 mediated postattachment inhibition at dose-dependent manners. They inhibited the HA pseudoviruses with mean IC_50_ values at 2.02 and 1.63 µg/ml, respectively. As control antibodies, AVFluIgG03 had no inhibitory activity on the attached viruses but CR6261 did, consistent with the previous reports [12], [19].

### Efficient Inhibition of Cell Fusion and Protease Susceptibility

**Figure 7:**
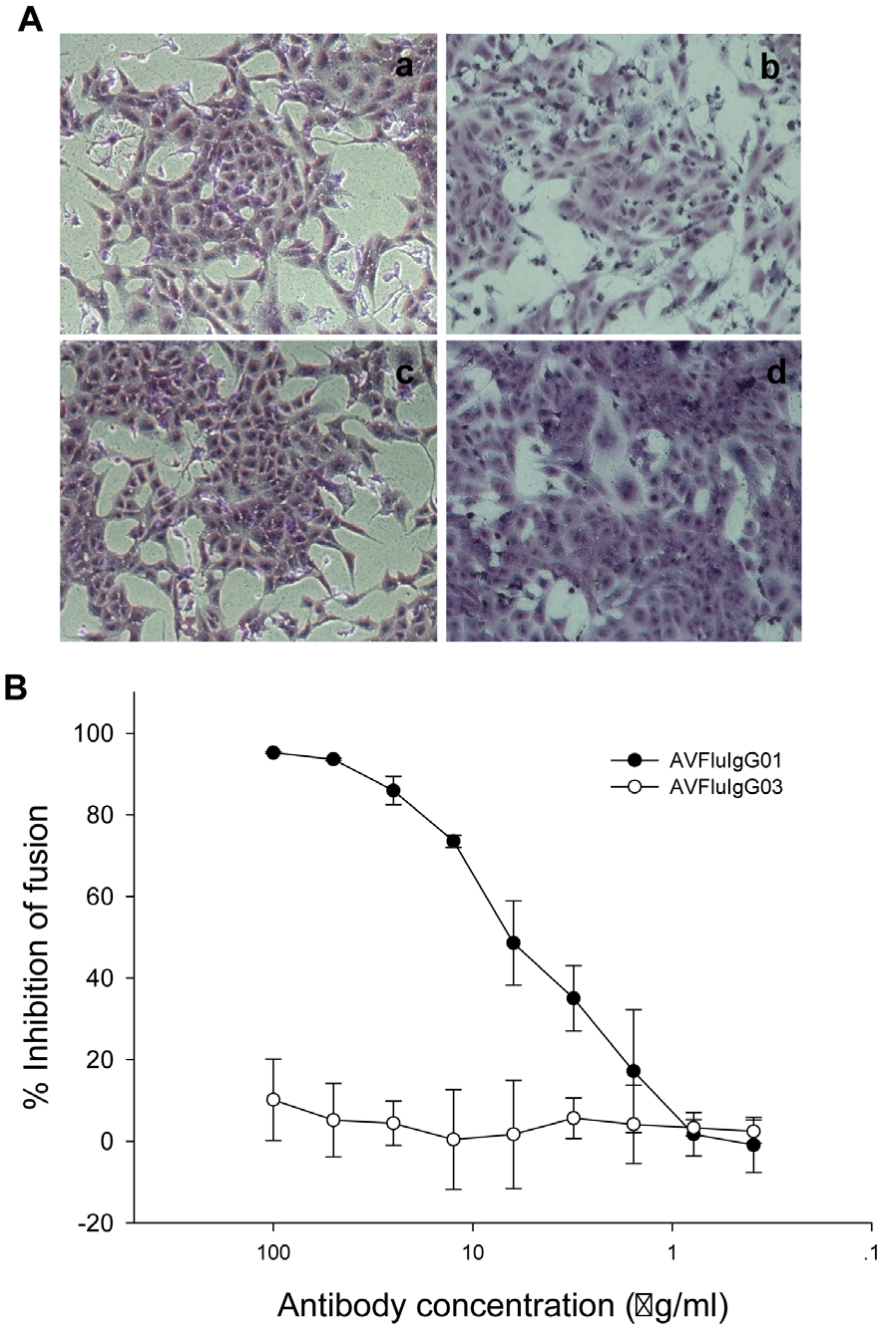
Inhibition of AVFluIgG01 on HA-mediated cell-cell fusion. A. AVFluIgG01 inhibits the low-pH induced cell-cell fusion in Vero cells that were transfected with the plasmid encoding Viet04 HA protein (pViet04-HA). The cells were stained with crystal violet. (a) Control cells (no HA transfection); (b) Positive control (without antibodies); (c) Treated with 50 µg/ml AVFluIgG01; (d) Treated with 50 µg/ml control mAb (AVFluIgG03). **B**. AVFluIgG01 inhibits cell fusion between 293T effector cells and MDCK target cells at a dose-dependent manner. 293T cells were co-transfected with pViet04-HA and pGAL4-VP16 and MDCK cells were transfected with pGal5-luc. After fusion induced by low-pH, the luciferase activity was measured in the cell lysates. The percent inhibition and IC_50_ were calculated.

As was revealed above by the postattachment neutralization assay, AVFluIgG01 might inhibit not only the receptor-binding step but also the membrane fusion step. To directly evaluate whether it is the case, we performed two cell fusion assays as described in the materials and methods. In the first fusion assay, the Vero cells were transfected with HA and induced for fusion by low-pH in the presence or absence of AVFluIgG01 or a control mAb. As shown in Fig. 7A, AVFluIgG01 did inhibit HA-mediated cell-cell fusion. In the second fusion assay, we demonstrated that AVFluIgG01 could inhibit the fusion between the HA-transfected 293T effector cells and MDCK target cells with a typical dose-response curve. As a control, AVFluIgG03 had no such effects in both systems.

**Figure 8:**
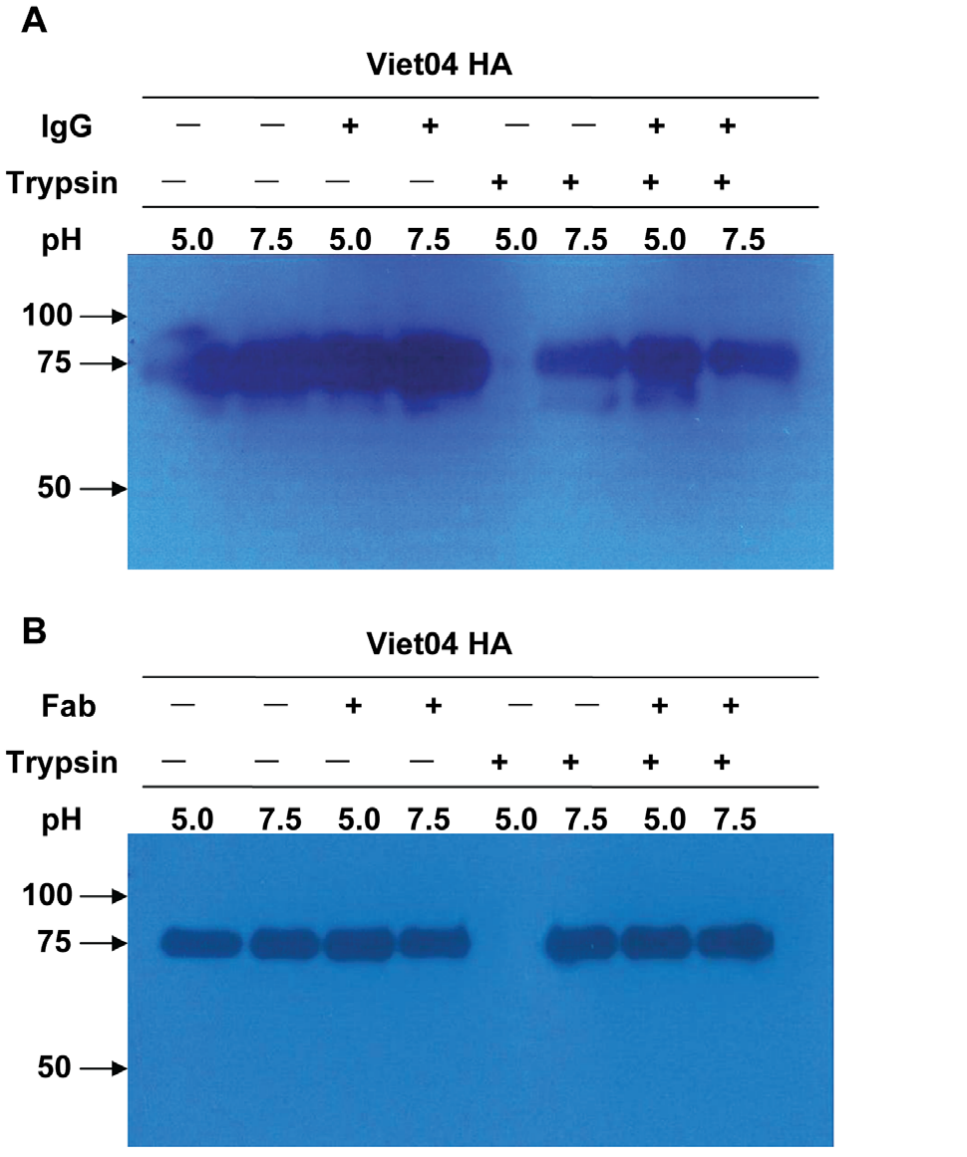
Inhibition of AVFluIgG01 on the pH-induced conformational changes of HA. The recombinant Viet04 HA proteins were incubated with or without AVFluIgG01 (**A**) or its Fab (**B**) at 37°C for 1 h. The mixture was either held at pH 7.5 (lanes 2, 4, 6 and 8) or changed to pH 5.0 (lanes 1, 3, 5 and 7) at 37°C for 1 h. After the pH was restored to 7.5, trypsin (1 µg/ml) was added to detect the acquisition of pH-induced protease susceptibility (lanes 5 to 8). The samples were then analyzed by western blotting. Molecular markers are given on the left.

AVFluIgG01 is a receptor-binding subunit (HA1)-specific mAb. Why could it also inhibit HA-mediated cell membrane fusion? We sought to ascertain its mechanism by a protease susceptibility assay, in which the HA protein was exposed to a low-pH to trigger a conformational change and acquire sensitivity to cleavage by trypsin. Convincingly, the low-pH treated HA could be completely digested by trypsin in the absence of AVFluIgG01 or its Fab fragment (lane 5 in Fig. 8A and 8B). However, both formats of antibodies were able to protect the HA from the degradation by protease (lane 7 in Fig. 8A and 8B). These results suggest that binding of AVFluIgG01 or its Fab fragment can block the HA to undergo a conformational change and thus render it resistant to proteolysis.

## Discussion

AVFluIgG01 is newly-isolated human mAb possessing potent and broad neutralizing activity against the highly pathogenic H5N1 influenza A virus [12]. The *in vivo* protection of AVFluIgG01 against challenge by H5N1 virus highlights its potential application as a therapeutic antibody or as a basis for vaccine development. In both cases, characterizations of its antigenic epitope and mechanism of neutralization is a key step. In the present study, we have first demonstrated that the epitope of AVFluIgG01 is a conformation-dependent and consists of three discontinuous sites. The site I comprises three amino acids residues (I116, I117, P118), while both of site II (W122, S123) and site III (Y164, T167) comprise two amino acids. In the 3D structure of H5N1 HA, these epitope residues are closely located at the tip of the membrane-distal globular head of HA and neighbored with the receptor-binding domain, which comprises the 190-helix (HA1 188 to 190), 130-loop (HA1 134 to 138) and 220-loop (HA1 221 to 228) as colored in red in Fig. 4B. Interestingly, although this region is highly variable but all the epitope participatory residues are highly conserved among diverse H5 clades and subclades. We believe that this conservation could provide the basis for antibody to exert its broad-spectrum anti-H5N1 activity.

Obviously, our present results differ from the previous data reported by Sun *et al* [12]. The authors considered that the AVFluIgG01 was directed against a linear epitope based on its reactivity in the Western-blotting assay. The epitope was further located within the amino acid sequence of ^116^IIPKSSWSS^124^ based on the mutagenesis analysis. In our Western blotting assays using both recombinant proteins and transfected cells as samples, AVFluIgG01 did repeatedly react with the HA protein in the gels (data not shown). In general view, the simplest way to determine whether an epitope is conformational or linear is by immunoblotting after SDS-PAGE [20]. If the antibody still binds after the protein has been denatured, the epitope is unlikely to be highly conformational. However, protein renaturation can occur after SDS gel electrophoresis or after electroblotting, which may result in an epitope refolded. In particular, a positive reaction on immunoblots can also be achieved by non-linear epitopes where the reacting portions are in relatively close proximity. In our experiences, a panel of mouse mAbs against the spike protein of SARS-CoV reacted with antigens on immunoblots but their epitopes were conformation-dependent [21]–[23]. Therefore, the AVFluIgG01 epitope might be such the case. The steric residues in the three sites could be readily refolded through short-range interactions to keep a “local” conformation. But the epitope could not be refolded when the HA protein was treated by a reducing reagent followed by blocking the reactive sites, thus resulting in the loss of the reactivity in ELISA wells. Contradicting to the previous conclusion, AVFluIgG01 could not recognize any of the synthesized peptides that contain or overlap the proposed linear epitope sequence (^116^IIPKSSWSS^124^). Taken together, the data strongly suggested a conformation-dependent epitope for AVFluIgG01. Therefore, we decided to map its contacting residues on the surface of the HA tridimensional structure by using a random peptide phage display library in combination with the computational prediction and experimental verification.

Vaccination is considered to be the most efficient strategy to combat the influenza A virus, but its continous antigenic drift and occasional antigenic shift have significantly hampered the development of an universal influenza vaccine [24]–[25]. For many years, considerable efforts have been made to characterize the antigenic epitopes of the HA protein, but much of the available information pertains to antibodies generated from mice rather than humans [9], [26]. The frequent occurrence of highly pathogenic H5N1-infected human cases and the recent outbreak of a novel swine-like human H1N1 influenza virus have promoted the exploration of natural-occurring human antibodies as therapeutics or for vaccine design. In recent years, a number of human mAbs to H5N1 viruses have been generated from either immortalized human memory B cells obtained from patients who recovered from H5N1 disease or with combinational antibody library technologies [12], [17], [27]–[31]. It has been shown that some of these human antibodies possess broad-spectrum H5N1-neutralizing activity or cross-subtype reactivity to H1 viruses, and are highly effective in suppressing H5N1 virus in experimentally infected animals when administered prophylactically or therapeutically [12], [25], [28], [31]. Importantly, antigenic characterizations of the newly-isolated anti-H5N1 human mAbs have identified novel neutralizing epitopes in HA protein and provided intriguing insights for universal influenza vaccine development [12]–[13], [17], [19], [25], [28]. The best examples are human mAbs CR6261 and F10, which possess broadly neutralizing activity and are directed against the highly conserved epitopes in the HA2 subunit [19], [25], [28], [31]. It is also impressive that the epitope of AVFluIgG01 is overlapped by two other epitopes recognized by human mAbs (FLA5.10 and FLD21.140) that were isolated from the survivals of HPAI H5N1 in Vietnam [27]. FLA5.10 recognized a conformational epitope containing the residues ^115^QIIP^118^ and ^126^EASL^129^ and had a narrow (clade 1-specific) neutralization range, while FLD21.140 recognized a conformational epitope containing the residues ^121^SWS^123^ and ^164^YNNT^167^and had a relatively broader cross-clade neutralization than the former [13], [27]. During the preparation of this manuscript, a new anti-H5N1 human mAb, 65C6 with the neutralizing and protective activity has been reported by Hu *et al* [17]. It was isolated from a surviving patient from H5N1 infection in China, and mapped to a conformational epitope containing the residues P118, S121, K161, Y164 and T167 [17]. Given that these neutralizing human mAbs were isolated from different surviving H5N1-infected patients in independent laboratories but reacted with the overlapping epitopes that are closely located a similar region on the surface of HA 3D structure, we believe that the AVFluIgG01 epitope represents an immunodominant site during natural infection and can be used for immunogen design to elicit broad-spectrum neutralizing antibodies against highly pathogenic H5N1 viruses.

While the antigenic epitopes of influenza virus have been explored as a hotspot, mechanisms of neutralization by antibodies are paid with much less attention. Actually, both aspects are important for dissecting the structure and function of the HA protein and thus for developing immunotherapeutics and vaccines against influenza. In this study, we first demonstrated that AVFluIgG01 could block both HA-mediated virus attachment and postattachment steps, and then showed its direct inhibition on HA-mediated cell-cell fusion and acquisition of pH-induced protease sensitivity. The data suggest that AVFluIgG01 neutralizes the virus through inhibiting not only receptor-binding but also membrane fusion. In other word, both neutralization mechanisms can occur simultaneously. This may not be so surprising to know that this HA1-specific mAb can block two key steps for vial entry and infection. The previous studies demonstrated that some HA1-specific mouse mAbs against H1N1 (A/PR/8/34) behaved similarly, although their specificity was concentration-dependent event [32]–[33]. Interestingly, a recent study reported that HA1-specific mouse anti-H5N1 mAb 9F4 was not able to block the interaction between HA and its receptor but did block the virus entry at the postattachment step. Consistently, 9F4 was further shown to prevent the pH-mediated conformational change of HA. Indeed, the neutralization of influenza viruses is highly complex and multifaceted, and is governed by factors that include the virus strain, the antigenic epitope, the antibody affinity and concentration, and the cells used [32].

It was notable that the Fab fragment of AVFluIgG01 could not inhibit the receptor-binding step at a same concentration as its IgG molecule (Fig. 5). In a sharp contrast, it could efficiently block the membrane fusion step, as evidenced by its potent inhibition on the viral entry after cell attachment (Fig. 6) and on the HA acquisition of pH-induced protease sensitivity (Fig. 8). Previous studies established that some HA1-specific Fabs neutralize H1N1 viruses by inhibiting fusion activity only, differing from their IgGs [33]–[34]. On the other hand, some HA1-specific Fabs neutralize by inhibiting virus attachment only, but mechanism of neutralization by their IgGs is complex and occurs simultaneously through fusion inhibition and attachment inhibition [32]. Compared to HA1-specific mAbs, HA2-specific mAbs, such as CR6261, neutralize the virus by a mechanism of inhibiting membrane fusion activity exclusively [19], [28], [35]–[36].

In summary, the significance of the present study is threefold. First, it shows that AVFluIgG01 is directed against a novel and conserved conformational epitope in the globular head of HA protein. Second, the epitope residues have been finely mapped to three discontinuous sites that are closely neighbored with the receptor-binding domain. Third, AVFluIgG01 neutralizes the virus by a mechanism of inhibiting the receptor-binding and membrane fusion simultaneously. Our data have multiple implications for developing effective immunotherapeutics and vaccines against highly pathogenic H5N1 virus.

## Materials and Methods

### Antibodies, Peptides and Proteins

The generation and initial characterization of AVFluIgG01 and AVFluIgG03 were described previously [12]. Their recombinant IgGs or Fab fragments were produced in insect cells by the Sino Biological Inc, Beijing. CR6261, an human anti-HA2 mAb was similarly produced in insect cells according to the publications [31]. Mouse anti-HA2 (H5N1) mAb 9G1G9 was obtained from the Sino Biological Inc. HY498, an human anti-HIV gp120 Fab was generated in our laboratory and used as a control. A set of 77 overlapping peptides that span the entire sequence of the HA protein of H5N1 strain A/H5N1/Vietnam/1203/2004 (Viet04), range from 15–20 amino acids in length, were synthesized at the SciLight Biotechnology LLC, Beijing. Recombinant HA and HA1proteins derived from Viet04 (Viet04-HA) or A/Common magpie/Hong Kong/2256/2006 (HK06-HA) were purchased from the eENZYME LLC (Gaithersburg, MD).

### ELISA

Reactivity of human (AVFluIgG01, AVFluIgG03 or HY498) or mouse (9G1G9) mAbs with various HA antigens was determined by ELISA. Briefly, 1 µg/ml recombinant Viet04-HA or HA1 or 5 µg/ml peptides were used to coat 96-well microtiter plates (Costar, Corning, NY) in 0.1 M carbonate buffer (pH 9.6) at 4°C overnight. The plates were blocked with 3% bovine serum albumin (BSA) for 1 h at RT and then washed with PBS-0.05% Tween 20 (PBST). A tested human or mouse mAb (diluted at 10 µg/ml) was added into wells and incubated at 37°C for 1 h, followed by three washes with PBST. Bound antibodies were detected with HRP-conjugated goat anti-human or mouse IgG (Sigma, Aldrich) at 37°C for 1 h, followed by three washes. The reaction was visualized by addition of 3,3′,5,5′-tetramethylbenzidine (TMB) substrate (Sigma), and stopped by the addition of 2 M H_2_SO_4_. The absorbance at 450 nm was measured by an ELISA plate reader (Bio-Rad).

To determine the effect of disulfide bond reduction on the binding of anti-HA mAbs, an ELISA plate was coated with recombinant HK06-HA at a concentration of 1 µg/ml and then treated with dithiothreitol (DTT) at a concentration of 10 mM for 1 h at 37°C, followed by washes. The plate wells were then treated with 50 mM iodoacetamide for 1 h at 37°C. After washes, a standard ELISA was performed as described above.

### Biopanning and Selection of Random Peptide Library by AVFluIgG01

A random phage display library (Ph.D-12), wherein the displayed peptides (12-mer) are expressed fused to the N terminus of gIII protein was purchased from New England Biolabs (Beverly, MA). Affinity selection of the phage clones from the library was performed according to the manufacturer’s instructions. In brief, the microtiter wells were coated with 100 µg/ml AVFluIgG01 in 100 µl 0.1 M NaHCO_3_ buffer (pH 8.6) at 4°C overnight. The coated wells were blocked with 0.5% BSA in NaHCO_3_ buffer for at least 1 h at 4°C and then washed 6 times with TBST (TBS +0.1% Tween-20). The phage library (1×10^10^) was added into the coated wells and incubated at room temperature (RT) for 1 h. After washing the plates 10 times with TBST, bound phages were eluted with 0.2 M Glycine-HCl (pH 2.2) containing 1 mg/ml BSA and neutralized with 1 M Tris-HCl (pH 9.1). The eluted phages were amplified by infecting log-phage *Escherichia coli* ER2738, and then concentrated by polyethylene glycol (PEG) precipitation. The phages were titered and submitted to the second round of selection. After three rounds of selection, the selected phage clones were identified by single-strand DNA sequencing and target specificity were confirmed by phage ELISA. Briefly, the microtiter wells were coated with 100 µl of 2 µg/ml of AVFluIgG01 and blocked by 0.5% BSA. Affinity-selected phages were added to the wells and incubated at RT for 1 h. After three washes with TBST (0.5% Tween 20), HRP-conjugated murine anti-M13 antibodies (Sigma) were added to the wells and incubated at 37°C for 1 h. The wells were extensively washed with TBST and reacted with TMB substrate (Sigma). Absorbance at 450 nm was measured with an ELISA reader (Bio-Rad).

### Computational Methods

Two web-available computer programs, Mapitope [15] and Pep-3D-Search [16], were applied to predict the AVFluIgG01 epitope based on the selected peptides (mimotopes) from the random phage display library. The algorithm of Mapitope is based on the assumption that the epitope is separated into several amino acid pairs (AAP) contributing for the binding to the antibodies and the entire set of peptides is enriched with AAP which mimic the genuine epitope. In Pep-3D-Search, a promising ACO (Ant Colony Optimization) algorithm was proposed to search matching paths on an antigen surface with respect to the query mimotopes or a motif. Both tools are validated for mapping a conformation-dependent epitope based on the affinity-selected mimotopes.

### Site-directed Mutagenesis

The plasmid pViet04-HA encoding a full-length HA of H5N1 (A/H5N1/Vietnam/1203/2004) was constructed and used as a template. A panel of HA mutants (Table 1) were generated by site-directed mutagenesis using the QuikChange XL kit (Stratagene, La Jolla, CA). The primers used for construction of HA mutants were designed and synthesized according to the manufacturer’s instructions. The introduced mutations were verified by DNA sequencing.

### Flow Cytometry Analysis

The reactivity of AVFluIgG01 with wild-type (WT) HA or HA containing mutations was measured by flow cytometry. Briefly, 293T cells were transiently transfected with a specific plasmid. The transfected cells were harvested at 24 h after transfection and washed two times with PBS containing 1% BSA. AVFluIgG01 or a control mAb was added to the cells to a final concentration of 10 µg/ml and incubated at 4°C for 1 h. Cells were then washed three times with PBS containing 1% BSA and incubated with FITC-conjugated goat anti-human or anti-mouse IgG (Sigma) at 4°C for 1 h in the dark. After three washes, the cells were re-suspended in cold PBS and analyzed by Becton Dickinson FACScalibur (Becton Dickinson, Franklin Lakes, NJ).

### Immuofluorescence Assay (IFA)

The plasmids encoding HA (WT or mutants) were transiently expressed in 293T cells by transfection. After 24 hours, cells were directly stained in the wells. Briefly, the cells were first fixed by 4% paraformaldehyde in PBS (pH 7.4) for 15 min at RT, followed by three washes with PBS. The cells were then incubated with 1% BSA in PBST for 30 min to block unspecific binding of the antibodies. AVFluIgG01 or a control mAb was added to the cells at a final concentration of 10 µg/ml and incubated at RT for 1 h in a humidified chamber. After washes, bound antibodies were detected by using FITC-conjugated anti-human antibodies and observed under an immunofluorescence microscope.

### Virus Binding Inhibition Assay

The virus binding assay was performed using HA (Viet04)-pseudotyped HIV viruses as described previously [28], [37]. Briefly, the HA pseudoviruses were generated by co-transfection of 293T cells with the plasmid pViet04-HA, and a plasmid encoding NA protein of A/H5N1/Tailand/1(KAN-1)/2004 and a backbone plasmid encoding Env-defective, luciferaseexpressing HIV-1 genome (pNL4-3.luc.RE). The pseudoviruses were first incubated with tested antibody (AVFluIgG01 and its Fab fragment or control mAbs) diluted in DMEM containing 1% BSA at 4°C overnight. MDCK cells were seeded in 24-well plates and grew 24 h at a monolayer and then blocked with DMEM containing 1% BSA at 4°C for 1 h. The mixture of pseudoviruses and antibody was inoculated onto MDCK cells and incubated at 4°C for 2 h. Cells were rinsed four times with PBS containing 1% BSA to remove unbound viruses. Then, the cells were lysed in 250 µl of DMEM using a freeze-thaw method. The amount of cell-bound viruses in the presence or absence of antibodies was quantified by an HIV-1 p24 ELISA kit (Vironostika HIV-1 antigen microelisa system, Biomerieux). Percent inhibition and 50% inhibitory concentration (IC_50_) were calculated.

### Postattachment Neutralization Assay

The postattachment assay was performed as previously described [33], [37]. Briefly, HA-pseudoviruses were inoculated onto an MDCK monolayer in a 96-well plate at 4°C for 6 h. The unbound viruses were removed by washing with cold PBS. A tested antibody was serially diluted and added to the monolayer at 4°C for 2 h. The antibody was then removed by washing, and fresh DMEM was added to the monolayer before incubation at 37°C. After 72 h infection, the luciferase activity of the cells was measured by using the luciferase assay system (Promega, Madison) according to the manufacturer’s instructions. Percent inhibition and IC_50_ were calculated.

### Inhibition of Cell-cell Fusion

A cell fusion inhibition assay was performed as described previously [28]. In brief, Vero cells were grown at ∼90% confluent in 12-well plates and transfected with pViet04-HA plasmid (1.6 µg total DNA per well) using lipofectamine 2000 (Invitrogen) according to the instruction manual. After 24 hours of transfection, the culture medium was supplemented with 50 µg/ml of AVFluIgG01 or a control mAb for 1 h. After washing three times by PBS (pH 7.4), cells were incubated with low-pH fusion-inducing buffer (150 mM NaCl +10 mM HEPES, adjusted to pH 5.0) for 5 mins, and then returned to the standard culture medium for 2 hours at 37°C. Finally, cells were fixed with 4% polyoxymethylene and stained with 0.5% crystal violet for 20 mins.

To quantitatively analyze the inhibitory effect of AVFluIgG01 on HA-driven cell-cell fusion, a sensitive assay was adapted from our previous studies [38]–[39]. Briefly, 293T effector cells seeded in 48-well plates at 0.8×10^5^ cells per well were transfected with pViet04-HA in combination with plasmid pGAL4-VP16, which encodes the herpes simplex virus VP16 transactivator fused to the DNA binding domain of the *Saccharomyces cerevisiae* transcription factor GAL4. In parallel, MDCK target cells were transfected with plasmid pGal5-luc, which encodes the luciferase reporter gene under the control of a promoter containing five GAL4 binding sites. The day after transfection, effector cells were incubated with various concentrations of tested antibodies diluted in serum-free medium (OPTI-MEM)at 37°C for 1 h. After washes, the effector cells were treated with low-pH fusion inducing buffer (same as above) for 5 mins and added to the target cells. After co-culturing for an additional 48 h, the cells were lysed by cell culture lysis buffer, and the luciferase activity was measured by luciferase assay system (Promega). The percent inhibition and IC_50_ were calculated.

### Protease Susceptibility Assay

The protease susceptibility assay was adapted from the previous studies [19], [33], [37]. In brief, 2 µg of HA protein (Viet04) was incubated with AVFluIgG01 or its Fab at 37°C for 1 h. The neutralization mixture was then kept at pH 7.5 or lowered to pH 5.0 by addition of 0.1 M HCl and incubated at 37°C for 1 h. The pH of the mixture was then restored to neutrality (pH 7.5) by addition of 0.1 M Tris (pH 10). The change of the HA to the low-pH conformation was detected by incubation with trypsin (1 µg/ml) at 37°C for 1 h. Reaction was stopped by addition of non-reducing SDS loading buffer and was further analyzed by Western blotting. Briefly, the digested mixture was fractionated by 10% SDS-PAGE under reducing conditions. The separated proteins were electrotransferred and immobilized on a PVDF membrane. After blocked with 5% nonfat milk in PBS at 4°C overnight, the membrane was incubated with a mouse anti-HA mAb (specific for HA1) at 37°C for 1 h, followed by washes with PBS. HRP-conjugated goat anti-mouse IgG was added and incubated at RT for 1 h. The membrane was washed and incubated with ECL reagents (Pierce).
